# Cerebrospinal Fluid Soluble Tau aggregates as a Biomarker for Alzheimer’s disease

**DOI:** 10.1002/alz70861_108061

**Published:** 2025-12-23

**Authors:** Md Tohidul Islam, Thomas K Karikari, Kaj Blennow

**Affiliations:** ^1^ Göteborg Universitet, Gothenburg, Västra Gotland Sweden; ^2^ University of Pittsburgh, Pittsburgh, PA USA; ^3^ Clinical Neurochemistry Laboratory, Sahlgrenska University Hospital, Mölndal, Västra Götalands län Sweden; ^4^ Department of Psychiatry and Neurochemistry, Institute of Neuroscience and Physiology, The Sahlgrenska Academy, University of Gothenburg, Mölndal Sweden

## Abstract

**Background:**

Tau aggregation is a key pathological event in Alzheimer’s disease (AD), yet detecting tau aggregates in cerebrospinal fluid (CSF) remains challenging. We developed and validated an ultrasensitive CSF tau aggregate assay to evaluate its diagnostic performance in AD and its correlation with established AD biomarkers.

**Method:**

This cross‐sectional study utilized CSF samples from the TRIAD cohort, including cognitively unimpaired (CU), mild cognitive impairment (MCI), Alzheimer’s disease (AD), and non‐AD neurodegenerative disease controls. CSF tau aggregates were amplified using an in vitro seeding protocol, where samples (50 µL) were treated with pre‐formed tau aggregates and incubated for 30 hours at 37°C with shaking (450 rpm). Quantification was performed using a homogeneous immunoassay on the Simoa HD‐X platform, employing CT19.1 antibody (epitope: 331–361) for both capture and detection. CSF tau aggregate levels were compared with established AD biomarkers, including CSF *p* ‐tau181, *p* ‐tau217, total tau, neurofilament light (NfL), and tau PET SUVR.

**Result:**

We developed and validated an untrasensitive CSF immunoassy for tau aggrergates. A total of 225 participants were included. CSF tau aggregates were significantly higher in AD and MCI compared to CU and non‐AD groups. Strong correlations were observed between CSF tau aggregates and CSF *p* ‐tau217, *p* ‐tau181, and tau PET SUVR, supporting the pathological relevance of CSF tau aggregates in AD.

**Conclusion:**

This study demonstrates that CSF tau aggregates can serve as a fluid‐based biomarker for Alzheimer’s disease, showing strong correlations with established AD biomarkers, including CSF *p* ‐tau, total tau, and tau PET. Given that CSF is a more accessible biological fluid compared to the high cost and limited availability of tau PET, this assay provides a practical approach for detecting and monitoring tau pathology. While further studies are needed to determine its utility in preclinical stages, these findings suggest that CSF tau aggregate quantification may aid in AD diagnosis and staging, potentially capturing early pathological changes.

